# Immunohistochemical Evaluation of NOTCH1 Signaling Pathway in Oral Squamous Cell Carcinoma: Clinical and Prognostic Significance

**DOI:** 10.3390/ijms26189167

**Published:** 2025-09-19

**Authors:** Juan Carlos de Vicente, Paloma Lequerica-Fernández, Héctor Torres Rivas, Verónica Blanco-Lorenzo, Ana López-Fernández, Samuel Andrés Escalante-Narváez, Sergi Herrera i Nogués, Juan P. Rodrigo, Saúl Álvarez-Teijeiro, Juana M. García-Pedrero

**Affiliations:** 1 Department of Oral and Maxillofacial Surgery, Hospital Universitario Central de Asturias (HUCA), C/Carretera de Rubín, s/n, 33011 Oviedo, Asturias, Spain; samescalantemd@gmail.com (S.A.E.-N.);; 2 Instituto de Investigación Sanitaria del Principado de Asturias (ISPA), Instituto Universitario de Oncología del Principado de Asturias (IUOPA), Universidad de Oviedo, C/Carretera de Rubín, s/n, 33011 Oviedo, Asturias, Spain; palomalequerica@gmail.com (P.L.-F.); ana_lopez23@hotmail.com (A.L.-F.); jprodrigo@uniovi.es (J.P.R.); saul.teijeiro@gmail.com (S.Á.-T.); 3 Department of Surgery, School of Medicine, University of Oviedo, 33006 Oviedo, Asturias, Spain; 4 Department of Biochemistry, Hospital Universitario Central de Asturias (HUCA), C/Carretera de Rubín, s/n, 33011 Oviedo, Asturias, Spain; 5 Department of Pathology, Hospital Universitario Central de Asturias (HUCA), C/Carretera de Rubín, s/n, 33011 Oviedo, Asturias, Spain; ress_444@yahoo.com (H.T.R.); veronica.blanco@sespa.es (V.B.-L.); 6 Department of Otolaryngology, Hospital Universitario Central de Asturias (HUCA), C/Carretera de Rubín, s/n, 33011 Oviedo, Asturias, Spain; 7 Centro de Investigación Biomédica en Red de Cáncer (CIBERONC), Instituto de Salud Carlos III, Av. Monforte de Lemos, 3-5, 28029 Madrid, Spain

**Keywords:** NOTCH1, HES1, p21, oral squamous cell carcinoma, prognosis, epithelial–mesenchymal transition

## Abstract

The aim of this study was to investigate the clinical and prognostic significance of the NOTCH1 pathway in oral squamous cell carcinoma (OSCC). To this end, the expression of NOTCH1 and two downstream targets, HES1 and p21, was evaluated by immunohistochemistry in 165 OSCC patient specimens. Clinicopathological associations and impact on survival were assessed. Possible mechanistic crosstalk with epithelial–mesenchymal transition (EMT) induction through combined E-cadherin and Vimentin markers, or mTORC1 activation by means of phospho-S6 expression were also investigated. NOTCH1 staining was detected in 56 (35%) tumors, nuclear HES1 in 131 (81%) and nuclear p21 in 116 (70%) tumors. p21 was strongly correlated with mTORC1 activation and HES1 expression was inversely associated with EMT status. NOTCH1 expression was positively associated with an advanced T stage, neck lymph node metastasis, advanced TNM stage, second primary cancer, and was significantly associated with shorter disease-specific survival (DSS). By contrast, HES1 and p21 expression showed significant associations with early clinical stages, and combined p21 and pS6 expression (p21+/p-S6+) distinguished good-prognosis patients. Multivariate Cox analysis further revealed NOTCH1 expression as a significant independent predictor of poor DSS. Mechanistically, we found a strong link between p21 and pS6 proteins, which could potentially serve as a good-prognosis classifier for OSCC patients.

## 1. Introduction

Oral squamous cell carcinoma (OSCC), the most frequent head and neck cancer, shows a five-year mortality rate close to 50% [1]. Poor prognosis is mainly attributed to local recurrence, lymphatic metastasis, and resistance to conventional therapy. This aggressive behavior might be caused by dysregulation of signaling pathways that control key biological processes implicated in tumorigenesis [2]. Moreover, there is an unmet need to identify biomarkers for prognosis assessment in OSCC as well as molecular targets able to offer more effective treatment options for this disease.

The NOTCH pathway is a ubiquitous conserved signaling system that plays important roles in cell-fate determination from embryonic life to throughout adulthood [3]. It also participates in relevant biological processes including cellular communication, proliferation, and differentiation [4]. NOTCH pathway is a juxtacrine signaling pathway initiated when one cell expressing the appropriate ligand (Jagged-1 and -2 of the Serrate family or Delta-like1, 3 and 4) interacts with another neighboring cell expressing one of the four NOTCH paralogs (NOTCH1-4). NOTCH1 is the most frequently expressed in human cancers [5]. The NOTCH1 protein is a transmembrane cell receptor. Upon ligand binding to its N-terminal domain, the transmembrane NOTCH1 receptor is cleaved twice [6,7], giving an intracellular fragment of NOTCH (NICD). Then, NICD translates into the nucleus where it interacts with DNA-binding protein CSL/CBF1/RBPjk, thereby recruiting coactivators MAML (mastermind-like) proteins to initiate transcription of target genes [3].

The most prominent NOTCH target genes are the hairy and enhancer of split (HES) and the hairy and enhancer of split related to YRPW motif (HEY) families, but also p21 (WAF1/Cip1/CDKN1A) [8] and Slug (Snail2) [9], among others. Pickering et al. [10] conducted the first genomic analysis of OSCC and found that Notch pathway was disrupted in 66% of cases. Moreover, HES1 has been found to be overexpressed in 32% of head and neck squamous cell carcinomas (HNSCC), but only in the absence of NOTCH1-inactivating mutation [6]. Previous studies showed NOTCH upregulation in OSCC, suggesting that NOTCH signaling could play a crucial role in oral oncogenesis [11,12,13,14,15,16,17,18]. Inactivating NOTCH1 mutations have also been detected in OSCC in 11–15% Caucasian patients [11,13,14,15] and more than 40% of Chinese patients [12], being the second most frequently mutated gene in HNSCC after *TP53* [11,14,15].

According to these data, the role of NOTCH in cancer remains controversial, and there is growing evidence for the dual function of NOTCH1 as both a tumor suppressor or an oncogene depending on the cellular and tissue context [13,19,20]. Thus, it has been demonstrated that NOTCH1 may inhibit tumor growth [21], but it could also play a part in early cancer development [21], probably by the promotion of epithelial–mesenchymal transition (EMT) [22].

EMT is a process in which epithelial tumor cells lose their adhesive phenotype and concomitantly acquire mesenchymal features, including high cell motility and invasive capacity that are critical to enable cancer metastasis [23]. Epithelial cadherin (E-cadherin) and Vimentin are two hallmarks of EMT, and E-cadherin loss and Vimentin upregulation have been associated with the presence of metastasis in HNSCC, including OSCC [24]. Wangmo et al. [25] distinguished three different EMT status subgroups by combining the expression of E-cadherin and Vimentin: (1) no EMT, defined as positive E-cadherin and negative Vimentin staining; (2) partial EMT, defined as either positive E-cadherin and positive Vimentin or negative E-cadherin and negative Vimentin expression, and (3) complete EMT, defined as negative E-cadherin and positive Vimentin expression.

Despite the pathobiological role of NOTCH1, it has been extensively studied in different malignancies, but its clinical significance has been scarcely investigated and it remains controversial in OSCC [21]. This prompted the present study aimed at ascertaining the clinical and prognostic significance of NOTCH1 expression and key downstream pathway targets in OSCC patients.

## 2. Results

### 2.1. Patient Characteristics

The main clinicopathological data are summarized in Table 1. The mean age was 63.8 years, 113 (68.5%) patients were men, 107 (65%) were smokers and 89 (54%) were alcohol drinkers. Thirty-seven (22.4%) tumors were stage I, 51 (30.9%) stage II, 30 (18.2%) stage III, and 47 (28.5%) were stage IV. The majority of tumors (105, 63.6%) were well differentiated. None of the patients underwent any treatment before surgery. Complementary radiotherapy and/or chemotherapy were administered in 66 (40%) and 18 (11%) cases, respectively.

The mean and median follow-up times were 62.58 and 59 months, respectively. At the end of the follow-up, 67 (40.6%) patients died of the OSCC. Tumor recurrence occurred in 82 (49.7%) cases. The mean and median follow-up times were 86.12 and 81 months, respectively, for patients without tumor recurrence, while these figures were, respectively, 37.6 and 13 months for recurrent patients. The five-year DSS and overall survival (OS) rates were 61% and 52%, respectively.

### 2.2. Immunohistochemical Evaluation of NOTCH1 Expression and Downstream Targets in OSCC Specimens

**Figure 1:**
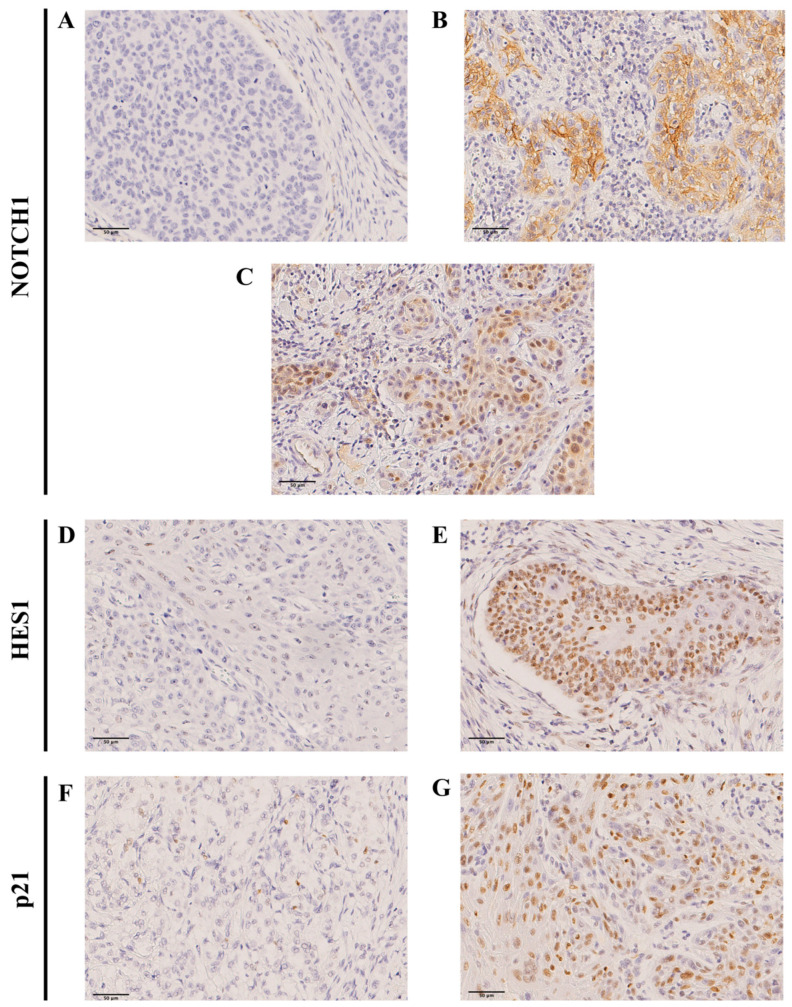
Immunohistochemical analysis of NOTCH1, HES1 and p21 in OSCC tissue specimens. Representative images of tumors showing (**A**) negative NOTCH1 staining, (**B**) positive cytoplasmic NOTCH1, (**C**) positive nuclear NOTCH1, (**D**) negative HES1 staining, (**E**) positive nuclear HES1, (**F**) negative p21 staining, and (**G**) positive nuclear p21 staining. Scale bar 50 µm.

NOTCH1 expression was successfully evaluated in 155 out of 165 OSCC samples. A total of 96 tumors (62%) showed negative expression, while the remaining 59 cases (38%) displayed cytoplasmic NOTCH1 expression (Figure 1A,B). In addition, nuclear NOTCH1 expression was detected in 7 (5%) cases (Figure 1C). HES1 expression was also successfully evaluated in 163 out of 165 tumor samples. HES1 immunostaining showed a nuclear pattern (Figure 1D,E), found in 131 (81%) out of 163 tumors. In addition, the expression of the NOTCH1 target gene p21 was also successfully evaluated in 155 out of 165 tumor samples. Nuclear p21 was detected in 125 (81%) out of 155 tumors (Figure 1F,G).

There was no significant correlation between the expression of nuclear NOTCH1 and the two downstream targets nuclear HES1 and p21. It is, however, noteworthy that all seven tumors harboring nuclear NOTCH1 staining exhibited nuclear HES1 staining. Analogously, no associations were observed between the expression of cytoplasmic NOTCH1, HES1, and p21.

Since we previously demonstrated an alternative mechanism of p21 expression regulation by the mTORC1/4E-BP1 pathway [26], we assessed the expression of phospho-S6 (p-S6) as a surrogate marker of mTORC1 activation. Thus, p-S6 (Ser235/236) was evaluated in 156 OSCC samples, and 106 (68%) tumors showed positive expression. There was a significant positive association between the expression of p21 and p-S6 (*p* = 0.04). Remarkably, 87 p-S6-positive cases (82%) also concomitantly harbored positive p21 protein expression.

### 2.3. Associations of NOTCH1 Expression and Downstream Targets with Clinicopathological Variables

The expression of cytoplasmic NOTCH1 was positively and significantly associated with an advanced T stage (*p* = 0.004), the presence of neck lymph node metastasis (*p* = 0.013), advanced TNM stage (*p* = 0.003), moderately or poorly differentiated tumors (*p* = 0.001), and with the presence of a second primary cancer in the oral cavity (*p* = 0.002) (Table 2). In marked contrast, HES1 expression was positively associated with smoking habit (*p* = 0.016), early pT classification (*p* = 0.001), and early clinical stage (*p* < 0.0001). Similarly, p21 expression was also significantly associated with early pT classification (*p* = 0.05), and early clinical stage (*p* = 0.04). No significant associations were observed between nuclear NOTCH1 staining and any clinical and pathological features studied (Table 2).

### 2.4. Impact of NOTCH1, HES1, and p21 on Patient Survival

**Figure 2:**
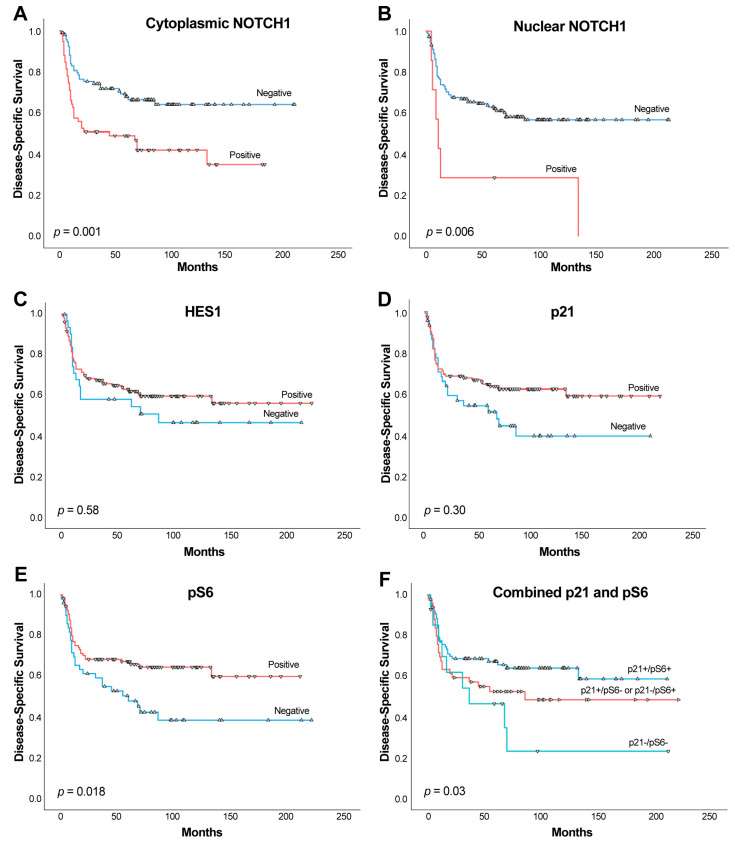
Kaplan–Meier disease-specific survival curves in the cohort of 165 OSCC patients categorized by (**A**) cytoplasmic NOTCH1 (positive versus negative), (**B**) nuclear NOTCH1, (**C**) nuclear HES1, (**D**) nuclear p21, (**E**) pS6 and (**F**) combined expression of p21 and p-S6 grouped as double-positive (p21+/p-S6+), single-positive (p21+/p-S6- or p21-/p-S6+), and double-negative cases (p21-/p-S6-). *p* values estimated by Log-rank test.

Kaplan–Meier analysis revealed that patients harboring cytoplasmic NOTCH1-expressing tumors showed a shorter DSS than those with negative expression (Log-rank test, *p* = 0.001, HR = 2.16; Figure 2A). Specifically, the mean survival time for patients with positive cytoplasmic NOTCH1 expression was 84.28 months (95% CI = 62.44 to 106.12 months), whereas for patients with negative cytoplasmic NOTCH1 was 145 months (95% CI = 125.74 to 164.25 months). Regarding nuclear NOTCH1-expressing tumors, a strong association with poorer DSS was also observed (Log-rank test, *p* = 0.006, HR = 3.06; Figure 2B). The mean survival time observed in patients harboring positive nuclear NOTCH1 tumors was 44.28 months (95% CI = 5.0 to 89.86 months), whereas in patients with negative nuclear NOTCH1, mean survival time was 130.04 months (95% CI = 113.96 to 146.13 months).

No significant associations were observed between the expression of HES1 and p21 and DSS (*p* = 0.58 and *p* = 0.30, respectively) (Figure 2C,D), although a tendency towards better DSS was observed. In addition, we found that p-S6 expression was significantly associated with better DSS (Log-rank test, *p* = 0.018, HR = 0.56; 95% CI = 0.34 to 0.91; Figure 2E). Taking into consideration our observation regarding the relationship between p21 and p-S6 expression, we next examined the impact of combined expression of both proteins on OSCC prognosis by using three combination subgroups: double-positive cases (p21+/p-S6+), single-positive cases (p21+/p-S6- or p21-/p-S6+), and double-negative cases (p21-/p-S6-). This allowed prognostic stratification of patients into three distinct subgroups. Thus, double-positive cases exhibited the best DSS (reference), single-positive cases an intermediate DSS (*p* = 0.18, HR = 1.45, 95% CI = 0.84 to 2.50) and double-negative cases showed the worst DSS (*p* = 0.006, HR = 2.52, 95% CI = 1.30 to 4.89) (*p* = 0.03, Figure 2F).

Finally, multivariate Cox analysis was performed including pT classification, neck lymph node metastasis, cytoplasmic and nuclear NOTCH1 expression, further revealing that the parameters independently associated with a poor DSS were T3–T4 classification (HR = 2.36, 95% CI 1.34 to 4.16, *p* = 0.003), the presence of neck lymph node metastasis (HR = 2.14, 95% CI 1.19 to 3.85, *p* = 0.01), and cytoplasmic NOTCH1 expression (HR = 1.82, 95% CI 1.05 to 3.14, *p* = 0.03); however, nuclear NOTCH1 expression did not retain its prognostic significance (HR = 1.78, 95% CI 0.70 to 4.53, *p* = 0.22).

### 2.5. Relationship Between the Expression of NOTCH1, HES1, and p21 and the EMT Status

The EMT status was evaluated by the immunohistochemical assessment of E-cadherin and Vimentin expression in our selected cohort of 165 OSCC patients. Cytoplasmic and nuclear NOTCH1 and nuclear p21 were not significantly associated with the expression of E-cadherin (*p* = 0.30, *p* = 1.0, and *p* = 0.62, respectively). A positive correlation was observed between cytoplasmic NOTCH1 and Vimentin expression (*p* = 0.017), whereas nuclear NOTCH1 and nuclear p21 were not significantly associated with Vimentin (*p* = 0.24, and *p* = 0.17, respectively). Additionally, HES1 expression showed significant associations with both E-cadherin and Vimentin proteins. Specifically, positive nuclear HES1 was observed in 80 (91%) tumors harboring positive E-cadherin staining and 50 tumors (68%) with negative E-cadherin staining (*p* < 0.0001). On the other hand, positive nuclear HES1 was found in 85 tumors (87%) with negative Vimentin staining and 45 cases (70%) with positive Vimentin, thereby showing an inverse significant association (*p* = 0.01).

According to these findings, neither cytoplasmic/nuclear NOTCH1 nor p21 were significantly associated with the EMT status (*p* = 0.49, *p* = 0.61, and *p* = 0.15). However, HES1 expression showed a strong inverse correlation with the EMT status (*p* = 0.001) (Table 3). Thus, positive nuclear HES1 was observed in 57 tumors (92%) classified as no EMT (i.e., E-cadherin +/Vimentin −), 51 tumors (80%) with a partial EMT and 23 tumors (62%) with complete EMT (i.e., E-cadherin −/Vimentin +).

Even though NOTCH1 expression was not significantly correlated with EMT (Table 3), we observed an increase in cytoplasmic NOTCH1 positivity with EMT. This observation prompted us to explore whether the poor prognosis of NOTCH1-expressing tumors could be linked to EMT, which is commonly associated with aggressive phenotypes and unfavorable clinical outcomes. As shown in Table 4, patients harboring NOTCH1-negative tumors exhibit the highest mean survival times for both DSS and OS (144.27 months and 118.66 months, respectively), whereas the prognosis for patients with NOTCH1-positive tumors worsened progressively with increasing EMT status (*p* = 0.01 and *p* = 0.03, respectively). Thus, patients harboring NOTCH1-positive tumors with complete EMT showed the worst DSS (mean = 70.48 months; 95% CI: 27.28–113.68; HR = 2.75; *p* = 0.008) and the worst OS (mean = 65.20 months; 95% CI: 24.94–105.46; HR = 2.01; *p* = 0.04).

Regarding nuclear NOTCH1 expression, only one case (0.6%) exhibited complete EMT, and two cases showed partial EMT. Therefore, the limited sample size precludes the possibility of conducting a reliable survival analysis.

## 3. Discussion

Dysregulation of NOTCH and/or downstream target genes have been widely detected in the development and progression of different human cancers. In this study, we specifically examined NOTCH1 protein expression in OSCC patient specimens and observed a frequency of 35%, which is similar to that reported by Cierpikowski et al. [27] who found NOTCH1 expression in 25% of OSCC samples. Furthermore, we observed in our series a relationship between NOTCH1 expression and an advanced T classification and TNM stage, as well as with neck lymph node metastasis and moderately–poorly differentiation, findings that are partially consistent with those of Osathanon et al. [17] and Yoshida et al. [28]. However, Cierpikowski et al. [27] and Wu-Chou et al. [29] failed to show any association between NOTCH1 expression and clinicopathological parameters. Moreover, Shah et al. [5] and Zhang et al. [16] reported that NOTCH1 expression was most frequently detected in oral cancers with neck lymph node metastasis, which is also in good agreement with our findings.

Regarding its prognostic significance, some authors described a relationship between increased NOTCH1 expression and worse survival, whereas others found an association between high NOTCH1 expression and a better prognosis, and even a lack of prognostic significance of NOTCH1 [27,30]. Lin et al. [31] reported that high NOTCH1 expression was associated with poorer survival in both OSCC and oropharyngeal carcinomas. Similarly, NOTCH1 expression has also been associated with worse overall survival in laryngeal cancer [32]. Conversely, NOTCH1 expression has been positively associated with a better survival in oropharyngeal cancer, regardless HPV status [33], and Grilli et al. [34] also found a significant association between NOTCH1 expression and better survival in a large HNSCC cohort of carcinomas of oropharynx, hypopharynx and larynx. In marked contrast, we herein demonstrated that the subgroup of OSCC patients harboring either cytoplasmic or nuclear NOTCH1-expressing tumors significantly showed a poorer disease-specific survival.

In order to explain the disparate results of NOTCH1 as prognostic factor we have to consider the biological role of this signaling pathway. Several studies have shown upregulation of NOTCH1 in OSCC [28,35] and it has been reported that 32% of HNSCC displayed an overexpression of downstream ligands of NOTCH1, whereas 15% have inactivating mutations and suppression of downstream ligands of NOTCH1 [14]. Additionally, NOTCH1 could also be involved in the early progression from potentially oral malignant lesions into OSCC [33]. These observations have raised the hypothesis that NOTCH may act as a suppressor gene in head and neck cancer [20,36,37], although experimental studies have provided evidence to the contrary [38]. The simplest explanation is that the role and impact of NOTCH is context-dependent to cell and tissue [13,19]. In addition, the clinical and biological significance of NOTCH1 could also be depend on the disease’s stage. Thus, in cervical cancer, NOTCH1 expression promotes tumorigenesis in early disease stages, whereas it exerts a tumor suppressive role in late disease stages [39]. Even though NOTCH1 undergoes inactivating mutations in at least the 10% HNSCC, including OSCC [11,13,14,15], a number of activating mutations have also been detected in OSCC [12,14,15]. Sun et al. [13] reported that NOTCH signaling pathway was active in about one third of Caucasian HNSCC patients. The lack of prognostic significance of NOTCH1 found by some authors along OSCC progression [27] might be explained at the light of the findings of Ding et al. [33] who indicated NOTCH1 to only be considered as an early biomarker for progression risk assessment of potentially oral malignant disorders into OSCC. Nevertheless, according to our findings NOTCH1 expression is also linked to advanced clinical stages thereby suggesting a role for this protein in OSCC progression. Therefore, beyond the potential role of NOTCH1 in early stages of oral tumorigenesis, it could also be involved in disease progression, thus acting as an oncogene rather than a tumor suppressor. Sun et al. [13] revealed a bimodal pattern of NOTCH pathway alterations in HNSCC, with a smaller subset of tumors harboring inactivating NOTCH1 receptor mutations, and a larger subset of tumors exhibiting NOTCH1 pathway activation. NOTCH pathway may influence in the epithelium through its target protein HES1, which promotes the cell differentiation known as the basal-to-spinous switch [40]. Our results suggest that after being activated in the normal epithelium NOTCH may remain activated in a subset of oral carcinomas contributing not only to tumorigenesis, but also to tumor progression.

Our study also provides relevant mechanistic insights of NOTCH1 pathway activation status in OSCC patients. Thus, NOTCH1 expression was detected in 35% of OSCC tissue specimens, the expression of the downstream target genes HES1 and p21 was found in 81% and 70% of tumors, respectively. In addition, all cases with nuclear NOTCH1 expression concomitantly showed HES1 positivity; however, there was no correlation between NOTCH1 and p21 expression. According to these findings, other mechanisms beyond NOTCH signaling should be responsible for the overwhelming frequent expression of HES1 and p21 proteins in our OSCC cohort. In this regard, we found a significant correlation between p21 and the mTORC1 activation marker phospho-S6, with 85% double-positive cases, which is in good accordance to our previous work unprecedentedly uncovering an alternative mechanism of p21 expression regulation by the mTORC1/4E-BP1 pathway that was operative in over 70% of HNSCC [26] as well as OSCC [30] and linked to a good prognosis [26,41]. In fact, double-positive cases pS6+/p21+ exhibited the best prognosis. Contrasting this, cytoplasmic and nuclear NOTCH1 consistently showed significant associations with a worse prognosis in our cohort of OSCC patients, and more importantly, cytoplasmic NOTCH1 expression was found an independent predictor of poor disease-specific survival. Altogether, these data indicate that p21 expression is predominantly regulated by mTORC1/4E-BP1 pathway rather than NOTCH1 signaling. Similarly, our immunohistochemical data also indicate that HES1 expression is uncoupled from NOTCH1 expression, thereby suggesting that other NOTCH1-independent mechanisms should be contributing to HES1 expression in over 80% of the studied OSCC specimens. Evidence for NOTCH1-independent HES1 expression has been demonstrated in Ewing’s sarcoma [42], neuroblastoma [43] and endothelial cells [44] through mechanisms that involve JNK signaling [44] or HIF-1-mediated hypoxia signaling [45], among others.

We also assessed whether NOTCH1 and HES1 could play a role in tumor progression and metastasis through EMT induction. It has been reported that the HES1 is a key gene in metastasis and drug resistance [46]. It has been demonstrated that NOTCH is able to promote EMT through E-cadherin repression by Slug (Snail-2) [47]. Moreover, HES1 was also found to significantly reduce E-cadherin expression and to also increase Vimentin expression [48]. Contrary to this, we found a significant inverse correlation between HES1 expression and EMT induction by joint detection of E-cadherin and Vimentin protein expression. Analogously, Wang et al. [49] observed that the loss of HES1 expression positively correlated with EMT in colon adenocarcinomas commonly harboring KRAS or BRAF mutation. Therefore, HES1 could exhibit distinct roles depending on the cancer type, tissue context or genetic background.

All these observations support the notion that NOTCH1 signaling pathway has dissimilar roles in oral carcinogenesis, and there could be a possible relevant crosstalk with other signaling pathways that may influence or alter the expression/activity, ultimately leading to varying clinical and biological significance of this pathway depending on the cell and tissue context.

Our study has some limitations that should be acknowledged. First, the use of tissue microarrays (TMAs) may not fully capture intratumoral heterogeneity, although this technique enables standardized, high-throughput evaluation across large patient cohorts. Second, the retrospective nature of the study may introduce inherent biases; thus, prospective validation in independent cohorts will be important to strengthen our findings. Third, while we observed significant associations between NOTCH1 expression, its subcellular localization, and the expression of downstream effectors such as p21 and HES1, further validation in larger and independent datasets will be required to confirm the robustness and generalizability of these observations.

Finally, we highlight that our results should be interpreted within the context of an IHC-based expression analysis. While IHC offers critical insights into both protein levels and spatial distribution, future studies integrating multi-omics and functional approaches will be valuable to further elucidate the underlying regulatory mechanisms.

## 4. Materials and Methods

### 4.1. Patients and Tissue Specimens

A total of 165 patients with histologically confirmed OSCC were surgically treated at the Hospital Universitario Central de Asturias between 1996 and 2007. This retrospective study was conducted following the ethical criteria of Declaration of Helsinki and approved by the Institutional Ethics Committee of the Hospital Universitario Central de Asturias and by the Regional CEIC from Principado de Asturias (date of approval 14th of May 2019; approval number: 136/19 for the project PI19/01255) for the use of histopathological and clinical material for research purposes. We retrieved clinical and histopathological information from the patients’ files; and pathology reports; which are summarized in Table 1. Written informed consent was obtained from all patients. The tumor histological grade was determined according to the WHO classification [50] and the clinical staging was assessed according to the eighth edition of the AJCC classification [51]. The clinical endpoint of this study was disease-specific survival (DSS), which was calculated as the time interval from the initial treatment to the date of death for the tumor.

### 4.2. Immunohistochemistry (IHC)

Tissue microarrays (TMAs) were constructed by collecting single-tissue cores (1 mm in diameter) from the most morphologically representative areas of formalin-fixed, paraffin-embedded (FFPE) tissue blocks provided by the Principado de Asturias BioBank (PT20/00161). Three cores were taken per patient tumor block. The TMAs were cut into 3-μm thick sections and dried on Flex IHC microscope slides (DakoCytomation, Glostrup, Denmark). Antigen retrieval was carried out using Envision Flex Target Retrieval solution, high pH (Dako, Glostrup, Denmark).

Staining was performed at room temperature on an automatic staining workstation (Dako Autostainer Plus, Dako, Glostrup, Denmark) with the following primary monoclonal antibodies: anti-NOTCH1 (clone D1E11; Cell Signaling, Danvers, MA, USA) at 1:400 dilution, anti-HES1 (clone D6P2U; Cell Signaling, Danvers, MA, USA) at 1:200 dilution, anti-p21 (clone 4D10; Leica Biosystems NCL-L-WAF-1, Newcastle Upon Tyne, UK) at 1:10 dilution, anti-phospho-S6 Ribosomal Protein (Ser235/236; Cell Signaling #2211, Danvers, MA, USA) at 1:200 dilution, anti E-cadherin (clone 36/E-cadherin; BD Biosciences, San Jose, CA, USA) at 1:4000 dilution, and anti-Vimentin (clone RV202; Abcam, Cambridge, UK) at 1:200 dilution. The antibody–antigen complex was visualized with the Dako EnVision Flex + Visualization System (Dako) and diaminobenzidine chromogen as substrate.

The IHC results were independently evaluated by three observers (VBL, HTR and JPR), blinded to clinical information. The immunopositivity and the subcellular localization of the staining were both considered. Scoring was based on staining intensity and the percentage of stained tumor cells, as previously reported [26,34,41,52]. Specifically, NOTCH1 and HES1 immunostaining were, respectively, scored from 0 to 2 if 0% to 10%, 11% to 50%, and >50% of tumor cells showed cytoplasmic NOTCH1 staining or nuclear HES1 staining. The staining intensity was scored from 0 to 2 scale (0 = negative, 1 = weak, 2 = strong). The raw data were then converted into an Immunoreactive Score (IRS) by multiplying the staining intensity and quantity scores. Theoretically, the scores could range from 0 to 4. For statistical purposes, these scores were dichotomized as negative expression (score 0) versus positive expression (scores 1–4). Since the NOTCH1 antibody recognizes both the full-length and the intracellular domain NICD, according to NOTCH1 function, staining into the nucleus was also separately evaluated as a surrogate of NOTCH1 activation. Nuclear NOTCH1 staining was scored in a binary fashion, as positive versus negative depending on the presence or absence of stained tumor cells. p21 and p-S6 immunostaining was dichotomized as negative expression (0–10% stained cells) versus positive expression (>10% stained tumor cells), according to the cut-off points previously established [26,41].

### 4.3. Statistical Analysis

Statistical analyses were carried out using SPSS software version 27 (IBM Co., Armonk, NY, USA). Chi-squared and Fisher’s exact tests were used to evaluate the relationship between categorical variables. Survival analyses were performed using the Kaplan–Meier method, and survival rates compared using the log-rank test. Univariate and multivariate Cox regression model was applied to calculate the hazard ratio (HR) and 95% confidence interval (95% CI), as well as to determine independent prognostic factors in the presence of other relevant covariates. All *p*-values were based on two-sided statistical analysis, and for all analyses a *p*-value less than 0.05 was considered to be statistically significant.

## 5. Conclusions

NOTCH1 protein expression was detected in 35% of OSCC patient specimens, which was significantly associated with advanced disease stages, neck lymph node metastasis, poor tumor differentiation, and a second primary tumor. More importantly, NOTCH1 expression was found an independent predictor of poor DSS, thereby supporting an oncogenic role in OSCC. In marked contrast, the expression of HES1 and p21 proteins was associated with early clinical stages, and overwhelmingly detected in over 70% of tumors, indicative of NOTCH-independent regulatory mechanisms. Furthermore, our study unraveled relevant mechanistic insights demonstrating a strong link between p21 protein expression and mTORC1 activation (by means of phospho-S6 staining), and also a significant inverse correlation between HES1 and EMT induction. Moreover, combined p21/pS6 staining showed potential as a good-prognosis classifier for OSCC patients.

## Figures and Tables

**Table 1:** Clinical and pathological characteristics of the cohort of 165 OSCC patients selected for study.

Variable	Number (%)
Age (year) (mean ± SD; median; range)	63.8 ± 12.65; 64; 30–92
Gender	
Men	113 (68.5)
Women	52 (31.5)
Tobacco use	
Smoker	107 (65)
Non-smoker	58 (35)
Alcohol use	
Drinker	89 (54)
Non-drinker	76 (46)
Location of oral squamous cell carcinoma	
Tongue	75 (45)
Floor of the mouth	34 (21)
Other sites within the oral cavity	56 (34)
Tumor status	
pT1	39 (25)
pT2	70 (44)
pT3	22 (14)
pT4	19 (12)
Unknown	8 (5)
Nodal status	
pN0	95 (57.6)
pN1-3	63 (38.2)
No neck dissection	7 (4.2)
Clinical stage	
Stage I	37 (22.4)
Stage II	51 (30.9)
Stage III	30 (18.2)
Stage IV	47 (28.5)
G status	
G1	105 (63.6)
G2	51 (30.9)
G3	9 (5.5)
Clinical status at the end of the follow-up	
Alive and without recurrence	77 (46.7)
Dead of index cancer	67 (40.6)
Lost or died of other causes (censored)	18 (10.9)
Second primary carcinoma	3 (1.8)

**Table 2:** Associations between the expression of NOTCH1, HES1 and p21 and the clinicopathological variables in a cohort of 165 OSCC patients.

Variable	Cytoplasmic NOTCH1 (%)	*p*	Nuclear NOTCH1 (%)	*p*	Nuclear HES1 (%)	*p*	Nuclear p21 (%)	*p*
	Negative Positive		Negative Positive		Negative Positive		Negative Positive	
Gender		0.29		0.67		0.20		0.76
Men	64 (59) 44 (41)		102 (95) 6 (5)		19 (17) 93 (83)		21 (20) 84 (80)	
Women	32 (68) 15 (32)		46 (98) 1 (2)		13 (26) 38 (74)		9 (18) 41 (82)	
Tobacco use		0.88				0.016		0.62
Smoker	64 (62) 40 (38)		97 (93) 7 (7)	0.06	15 (14) 91 (86)		18 (18) 81 (82)	
Non-smoker	32 (63) 19 (37)		51 (100) 0 (0)		17 (30) 40 (70)		12 (21) 44 (79)	
Alcohol use		0.61		0.69		0.07		0.85
Drinker	53 (60) 35 (40)		83 (94) 5 (6)		13 (15) 76 (85)		16 (19) 69 (81)	
Non-drinker	43 (64) 24 (36)		65 (97) 2 (3)		19 (26) 55 (74)		14 (20) 56 (80)	
pT		0.004				<0.001		0.05
pT1 + T2	73 (68) 34 (32)		104 (97) 3 (3)	0.34	13 (12) 99 (88)		16 (15) 90 (85)	
pT3 + T4	17 (43) 23 (57)		37 (93) 3 (7)		15 (35) 28 (65)		12 (29) 29 (71)	
pN		0.013						0.64
pN0	61 (69) 27 (31)		84 (96) 4 (4)	1.0	13 (14) 80 (86)	0.11	15 (17) 73 (83)	
pN1-3	30 (49) 31 (51)		58 (95) 3 (5)		15 (24) 48 (76)		12 (20) 48 (80)	
Clinical stage		0.003				<0.001		0.04
I + II	59 (73) 22 (27)		78 (96) 3 (4)	0.71	7 (8) 79 (92)		11 (14) 70 (86)	
III + IV	37 (50) 37 (50)		70 (95) 4 (5)		25 (32) 52 (68)		19 (26) 55 (74)	
G status		0.001				0.62		0.89
Well	70 (73) 26 (27)		91 (95) 5 (5)	1.0	18 (18) 85 (82)		19 (19) 79 (81)	
Moderate	22 (44) 28 (56)		48 (96) 2 (4)		12 (24) 39 (76)		10 (21) 38 (79)	
Poor	4 (44) 5 (56)		9 (100) 0 (0)		2 (22) 7 (78)		1 (11) 8 (89)	
Perineural invasion		0.15		1.0		0.69		0.68
No	89 (60) 58 (40)		140 (95) 7 (5)		30 (20) 124 (80)		28 (19) 118 (81)	
Yes	7 (88) 1 (12)		8 (100) 0 (0)		2 (22) 7 (78)		2 (22) 7 (78)	
Vascular invasion		0.67		0.24		0.34		0.62
No	93 (62) 56 (38)		143 (96) 6 (4)		32 (21) 124 (79)		28 (19) 120 (81)	
Yes	3 (50) 3 (50)		5 (83) 1 (17)		0 (0) 7 (100)		2 (29) 5 (71)	
Clinical status at the end of the follow-up								
Alive without recurrence	52 (73) 19 (27)	0.002	71 (100) 0 (0)	0.03	12 (16) 63 (84)	0.57	12 (17) 58 (83)	0.81
Dead of index cancer	31 (48) 34 (52)		59 (91) 6 (9)		16 (24) 51 (76)		15 (23) 51 (77)	
Censored	13 (77) 4 (23)		16 (94) 1 (6)		4 (22) 14 (78)		3 (18) 14 (82)	
Second primary cancer	0 (0) 2 (100)		2 (100) 0 (0)		0 (0) 3 (100)		0 (0) 2 (100)	

**Table 3:** Associations between the expression of NOTCH1, HES1 and p21 with the epithelial–mesenchymal transition (EMT) status.

EMT Status	Cytoplasmic NOTCH1	*p*	Nuclear NOTCH1	*p*	Nuclear HES1	*p*	Nuclear p21	*p*
	Negative Positive		Negative Positive		Negative Positive		Negative Positive	
No EMT * Partial EMT ** Complete EMT ***	41 (67%) 20 (33%) 34 (57%) 26 (43%) 21 (62%) 13 (38%)	0.49	57 (93%) 4 (7%) 58 (97%) 2 (3%) 33 (97%) 1 (3%)	0.69	5 (8%) 57 (92%) 13 (20%) 51 (80%) 14 (38%) 23 (62%)	0.001	16 (27%) 43 (73%) 9 (15%) 51 (85%) 5 (14%) 31 (86%)	0.15

* E-cadherin +/Vimentin −; ** E-cadherin +/Vimentin + or E-cadherin −/Vimentin −; *** E-cadherin −/Vimentin +.

**Table 4:** Univariate Cox analysis of disease-specific and overall survival in the cohort of 154 OSCC patients according to cytoplasmic NOTCH1 expression and the epithelial–mesenchymal transition (EMT) status.

Survival	Variable	Number of Cases	Survival Number (%)	Survival Time (Months) Mean (95% CI)	HR (95% CI)	*p*
DSS	NOTCH1 negative NOTCH1 positive, complete EMT NOTCH1 positive, partial EMT NOTCH1 positive, no EMT	95 13 26 20	64 (67.4) 4 (30.8) 11 (42.3) 10 (50.0)	144.27 (124.87–163.67) 70.48 (27.28–113.68) 81.58 (48.42–114.73) 79.50 (52.37–106.62)	1 (reference) 2.75 (1.30–5.79) 2.32 (1.25–4.30) 1.62 (0.79–3.30)	0.01 0.008 0.008 0.18
OS	NOTCH1 negative NOTCH1 positive, complete EMT NOTCH1 positive, partial EMT NOTCH1 positive, no EMT	95 13 26 20	52 (54.7) 3 (23.1) 8 (30.8) 8 (40.0)	118.66 (98.51–138.82) 65.20 (24.94–105.46) 69.06 (37.56–100.57) 70.23 (44.68–95.77)	1 (reference) 2.01 (1.00–4.02) 2.02 (1.16–3.52) 1.46 (0.77–2.77)	0.03 0.04 0.01 0.24

Abbreviations: DSS. disease-specific survival; OS, overall survival: HR, Hazard Ratio; CI, Confidence Interval. Eleven cases were not valuable for NOTCH1 and EMT status simultaneously.

## Data Availability

The data that support the findings of this study are available on request from the corresponding authors.

## Abbreviations

OSCC—Oralsquamous cell carcinoma; DSS—Disease-specific survival; EMT—Epithelial-mesenchymal transition; FFPE—Formalin-fixed, paraffin-embedded; TMAs—Tissue microarrays; IHC—Immunohistochemistry; NICD—Notch intracellular domain; CSL/CBF1/RBPjk—DNA-binding protein involved in NOTCH signaling; MAML—Mastermind-like proteins; HEY—Hairy/enhancer-of-split related with YRPW motif; CDKN1A—Cyclin-dependent kinase inhibitor 1A (p21); HNSCC—Head and neck squamous cell carcinoma; p-S6—Phosphorylated S6 ribosomal protein; mTORC1—Mechanistic target of rapamycin complex 1; 4E-BP1—Eukaryotic translation initiation factor 4E-binding protein 1; HR—Hazard ratio; CI—Confidence interval; AJCC—American Joint Committee on Cancer.
